# Dithiolopyrrolone Natural Products: Isolation, Synthesis and Biosynthesis

**DOI:** 10.3390/md11103970

**Published:** 2013-10-17

**Authors:** Zhiwei Qin, Sheng Huang, Yi Yu, Hai Deng

**Affiliations:** 1 Key Laboratory of Combinatory Biosynthesis and Drug Discovery (Ministry of Education), School of Pharmaceutical Sciences, Wuhan University, Wuhan 430071, China; E-Mails: r01zq11@abdn.ac.uk (Z.Q.); hs19870604@163.com (S.H.); 2 Marine Biodiscovery Centre, Department of Chemistry, University of Aberdeen, Aberdeen AB24 3UE, Scotland, UK

**Keywords:** dithiolopyrrolone natural products, chemical isolation, total synthesis, biosynthesis, mode of action

## Abstract

Dithiolopyrrolones are a class of antibiotics that possess the unique pyrrolinonodithiole (4*H*-[1,2] dithiolo [4,3-*b*] pyrrol-5-one) skeleton linked to two variable acyl groups. To date, there are approximately 30 naturally occurring dithiolopyrrolone compounds, including holomycin, thiolutin, and aureothricin, and more recently thiomarinols, a unique class of hybrid marine bacterial natural products containing a dithiolopyrrolone framework linked by an amide bridge with an 8-hydroxyoctanoyl chain linked to a monic acid. Generally, dithiolopyrrolone antibiotics have broad-spectrum antibacterial activity against various microorganisms, including Gram-positive and Gram-negative bacteria, and even parasites. Holomycin appeared to be active against rifamycin-resistant bacteria and also inhibit the growth of the clinical pathogen methicillin-resistant *Staphylococcus aureus* N315. Its mode of action is believed to inhibit RNA synthesis although the exact mechanism has yet to be established *in vitro*. A recent work demonstrated that the fish pathogen *Yersinia ruckeri* employs an RNA methyltransferase for self-resistance during the holomycin production. Moreover, some dithiolopyrrolone derivatives have demonstrated promising antitumor activities. The biosynthetic gene clusters of holomycin have recently been identified in *S. clavuligerus* and characterized biochemically and genetically. The biosynthetic gene cluster of thiomarinol was also identified from the marine bacterium *Pseudoalteromonas* sp. SANK 73390, which was uniquely encoded by two independent pathways for pseudomonic acid and pyrrothine in a novel plasmid. The aim of this review is to give an overview about the isolations, characterizations, synthesis, biosynthesis, bioactivities and mode of action of this unique family of dithiolopyrrolone natural products, focusing on the period from 1940s until now.

## 1. Introduction

There is an urgent need for new antibiotics with novel cellular targets. Though resistance to existing antibiotics is increasing at an alarming rate, only four new structural classes of antibiotics have been introduced to the clinic in the last 50 years [1,2,3]. Dithiolopyrrolones are a group of potent antibiotic natural products that have been found in both Gram-negative and Gram-positive bacteria. They consist of a unique pyrrolinonodithiole (4*H*-[1,2] dithiolo [4,3-*b*] pyrrol-5-one) chromophore [4]. Since the isolation of the first member of this family aureothricin (**1**) from a soil bacterium *Streptomyces* sp*.* 26A over 65 years ago [5], this class of molecules has intrigued numerous research groups not only for their unique chemical structures and their antibacterial/antifungal activities but also the chemical logic and regulation of the biosynthesis. Many members of this family have already showed strong broad-spectrum activities towards Gram-positive and Gram-negative bacteria, Yeast, Fungi and even parasites [6]. Holomycin (**9**) appeared to inhibit the rafamycin-resistant bacteria. It also acts as antibacterial agent toward clinical pathogen methicillin-resistant *Staphylococcus aureus* N315. Its mode of action has been long attributed to inhibit the activity of bacterial RNA polymerase although the exact mechanism remained to be elucidated *in vitro*. In the last two decades, there has been an increasing interest in both synthetic and pharmacological investigations of this unique class of molecules due to the emerging significance of aryl-containing dithiolopyrrolone as antiproliferative agents [7].

Despite increasing attention in this rare class of antibiotic natural products, there has been no literature to summarize and critically evaluate the scientific conclusions throughout the studies on dithiolopyrrolones. This review will give an overview about the discovery and bioactivity, synthesis and biosynthesis of this family of rare natural products, covering the period since 1948. Table 1 provides a summary of the structures of naturally occurring dithiolopyrrolones that were identified so far.

**Table 1:** A summary of naturally occurring dithiolopyrrolone antibiotics. 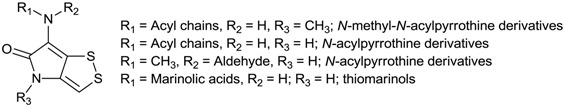

NO.	Name	Structure			Source	Ref.
		R1	R2	R3		
**1**	Aureothricin	CH_3_CH_2_CO	H	CH_3_	*Streptomyces* sp. 26A	[5]
**2**	Thiolutin	CH_3_CO	H	CH_3_	*Streptomyces albus*	[6]
**3**	Isobutanoylpyrrothine	(CH_3_)_2_CHCO	H	CH_3_	*Saccharothrix algeriensis*	[8]
**4**	Butanoylpyrrothine	CH_3_(CH_2_)_2_CO	H	CH_3_	*Saccharothrix algeriensis*	[9,10]
**5**	Senecioylpyrrothine	(CH_3_)_2_C=CHCO	H	CH_3_	*Saccharothrix algeriensis*	[9,10]
**6**	Tigloylpyrrothine	(CH_3_)CH=C(CH_3_)CO	H	CH_3_	*Saccharothrix algeriensis*	[9,10]
**7**	Xenorhabdin 4	CH_3_(CH_2_)_4_CO	H	CH_3_	*Xenorhabdus nematophilus* XQ1 (ATCC 39497)	[11]
**8**	Xenorhabdin 5	(CH_3_)_2_CH(CH_2_)_3_CO	H	CH_3_	*Xenorhabdus nematophilus* XQ1 (ATCC 39497)	[11]
**9**	Holomycin	CH_3_CO	H	H	*Streptomyces griseus* (NRRL 2764)	[12]
**10**	*N*-Propanoylholothine	CH_3_CH_2_CO	H	H	*Streptomyces* sp. P662	[13]
**11**	vD844	CHO	CH_3_	H	*Actinomycete* sp.	[14]
**12**	Xenorhabdin 1	CH_3_(CH_2_)_4_CO	H	H	*Xenorhabdus nematophilus* XQ1 (ATCC 39497)	[11]
**13**	Xenorhabdin 2	(CH_3_)_2_CH(CH_2_)_3_CO	H	H	*Xenorhabdus nematophilus* XQ1 (ATCC 39497)	[11]
**14**	Xenorhabdin 3	CH_3_(CH_2_)_6_CO	H	H	*Xenorhabdus nematophilus* XQ1 (ATCC 39497)	[11]
**15**	Xenorhabdin 8	decanoyl	H	H	*Pseudoalteromonas* sp. SANK 73390	[15]
**16**	Xenorhabdin 9	dodecanoyl	H	H	*Pseudoalteromonas* sp. SANK 73390	[15]
**17**	Xenorhabdin 10	*E*-dec-3-enoyl	H	H	*Pseudoalteromonas* sp. SANK 73390	[15]
**18**	Xenorhabdin 11	*Z*-dec-4-enoyl	H	H	*Pseudoalteromonas* sp. SANK 73390	[15]
**19**	Xenorhabdin 12	*E*-tetradecenoyl	H	H	*Pseudoalteromonas* sp. SANK 73390	[15]
**20**	Xenorhabdin 13	*Z*-hexadecenoyl	H	H	*Pseudoalteromonas* sp. SANK 73390	[15]
**21**	Thiomarinol A	Marinolic acids A	H	H	*Pseudoalteromonas* sp. SANK 73390	[16]
**22**	Thiomarinol B	Marinolic acids B	H	H	*Pseudoalteromonas* sp. SANK 73390	[17]
**23**	Thiomarinol C	Marinolic acids C	H	H	*Pseudoalteromonas* sp. SANK 73390	[17]
**24**	Thiomarinol D	Marinolic acids D	H	H	*Pseudoalteromonas* sp. SANK 73390	[18]
**25**	Thiomarinol E	Marinolic acids E	H	H	*Pseudoalteromonas* sp. SANK 73390	[18]
**26**	Thiomarinol F	Marinolic acids F	H	H	*Pseudoalteromonas* sp. SANK 73390	[18]
**27**	Thiomarinol G	Marinolic acids G	H	H	*Pseudoalteromonas* sp. SANK 73390	[18]

## 2. Isolation and Characterization

The family of dithiolopyrrolonenatural products can be divided into three subfamilies: *N*-methyl, *N*-acylpyrrothine (thiolutin type), *N*-acylpyrrothine (holomycin type) and thiomarinol, a distinct group of PKS-NRPS hybrid antibiotics. In this section, the isolation and structural elucidation will be summarized.

### 2.1. *N*-Methyl, *N*-Acylpyrrothine (Thiolutin-Type) Derivatives

**Figure 1:**
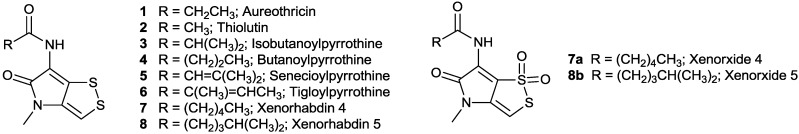
*N*-methyl, *N*-acylpyrrothine derivatives.

The first dithiolopyrrolone natural product, aureothricin (**1**), was reported in 1948 (Figure 1) [5]. Umezawa and co-workers isolated a new strain *Streptomyces* sp. 26A from a soil sample, collected in Mitaka Tokyo, Japan. Subsequently, they found the strain showed a new antibacterial spectrum and a yellow crystalline antibiotic substance was extracted. Two years later, the antibiotic thiolutin (**2**) was isolated by a research team in Pfizer, from a soil bacterium *Streptomyces albus* and described as a neutral, optically inactive, yellow-orange substance which appeared to resemble **1** at that time (Figure 1) [6]. Accordingly, the arranged interchange of the substances between the two research groups led to a conclusion that both compounds belong to the same family of antibiotics but are differentiated from their molecular formulas. The empirical formula of C_8_H_8_N_2_O_2_S_2_ and C_9_H_10_N_2_O_2_S_2_ for **1** and **2**, respectively, were proposed in 1952 (Figure 1) [19]. Both substances were of great interest at that time because of their high activity against a variety of fungi, ameboid parasites, Gram-positive, Gram-negative and acid fast bacteria [20].

Further study of UV absorption spectrum and chemical degradation [21] led to the elucidation of the structure of **2** to be an acetamide of 6-amino-4,5-dihydro-4-methyl-5-oxo-1,2-dithiolo[4,3-*b*]pyrrole. Accordingly, **1** was proposed to be the 3-propionamido derivative of **2**, which only differs from the length of acyl moiety in **2** (Figure 1).

Since then, **1** and **2** were repeatedly discovered from various actinomycete strains [22,23]. Isobutanoylpyrrothine(ISP) (**3**) (Figure 1) was first isolated from *Streptomyces pimprina* along with **1**, **2** and a polyene (heptaene) [8]. More recently, the rare actinomycete strain *Saccharothrix algeriensis* (NRRL B-24137) isolated from a south Algerian soil sample has been found to produce at least five dithiolopyrrolone antibiotics including **2** and four other derivatives of **2**, isobutanoylpyrrothine(ISP) (**3**), butanoylpyrrothine(BUP) (**4**), senecioylpyrrothine(SEP) (**5**) and tigloylpyrrothine(TIP) (**6**) (Figure 1) [9,10]. **3**–**6** contain the same chromophore of pyrrothine but differ from the acyl groups. The same research group also found that addition of organic acids into the semi-synthetic media influenced the yield of these dithiolopyrrolones in *S. algeriensis* [24]. The production of dithiolopyrrolones depends upon the nature and concentration of the organic acids in the culture medium.

Gram-negative bacteria such as symbiotic bacteria *Xenorhabdus* [11,25] were also found to produce thiolutin-type of dithiolopyrrolone natural products. In 1991, McInerney and co-workers [11] discovered two new *N*-methylated dithiolopyrrolone compounds (Figure 1), xenorhabdin 4 (**7**) and xenorhabidin 5 (**8**), from the culture broth of Xenorhabdus*nematophilus* XQ1 (ATCC 39497), along with other three des-*N*-methylated analogues **12**, **13** and **14** (see next section). *X*. *bovienii* is the only Xenorhabdus species that was found to produce oxidized xenorxide derivatives, **7a** and **8a **(Figure 1) [26]. *Xenorhabdus* are symbiotic enterobacteria associated with insect pathogenic, soil-dwelling nematodes of the families *Heterorhabditidae* and *Steinernematidae* [27,28]. It is believed that they are carried monoxenically within the intestine of the infective stage of the nematode. After invading the host insect, the nematodes release a toxin and an inhibitor of the insect immune system, as well as releasing *Xenorhabdus* and other symbionts. The bacterial symbionts, in turn, provide nutrients to the nematodes and produce antibiotics which inhibit the growth of other microbial flora in the insect cadavers. Intriguingly, *Xenorhabdus* *nematophilus* has two growth phases when cultured in the lab but only phase one metabolites, including Xenorhabdins, possess a wide spectrum of antibiotic activity.

### 2.2. *N*-Acylpyrrothine (Holomycin Type) Derivatives

**Figure 2:**
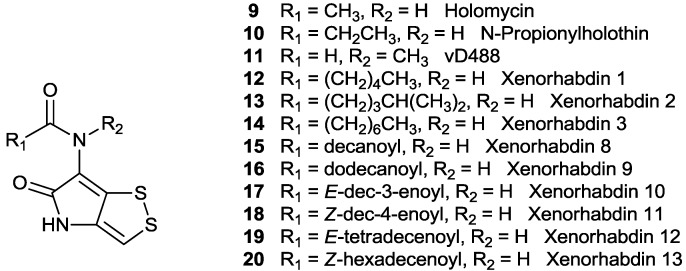
*N*-acylpyrrothine derivatives.

Holomycin **9** (Figure 2) is a des-*N*-methylthiolutin and was first identified in 1961 from the culture broth of a new strain of *Streptomyces griseus* (NRRL 2764), isolated from a soil sample at Riccino, Italy [12]. Although **9** is closely related to **2**, these two compounds differ from the physical and chemical properties, such as melting points, IR spectrum and behavior under paper chromatographic examination. Later on, holomycin and *N*-propionyl derivative **10** (Figure 2) were isolated from mutant strains of *Streptomyces* sp. P662 [13] and *Streptomyces clavuligerus* [29]. Interestingly, the wild types of these two *Streptomyces* strains are also producers of cephamycin C, a potent β-lactam antibiotic, which is biologically synthesized from aminoadipic acid, cysteine and valine [29,30,31]. The wild type *Streptomyces* sp. P6621 was found to produce cephamycin C [32] but does not produce **9** and **10**. Chemical mutagenesis led to generate the mutant *Streptomyces* sp. P6621-7N49 that only produces half the amount of cephamycin C with the production of **9** and **10** [32]. It was proposed that the production of **9** and **10** decrease the pool of cysteine available for cephamycin C biosynthesis and thus diminishes the level of cephamycin C produced. *Streptomyces clavuligerus* ATCC27064 has capacity to produce two clinically important antibiotics, the β-lactam antibiotic cephamycin C [33] and the β-lactamase inhibitor clavulanic acid [34]. Similar to the above case, the production of holomycin **9** in the wild type *S. clavuligerus* is not detectable. The mutant strain IT1, generated by UV mutagenesis of the parent strain of *S. clavuligerus,* led to overproduction of holomycin [29]. It was proposed that the unstable genetic element affect the production of holomycin [35]. Holomycin was also found from marine *Streptomyces* sp. M095 which was isolated from a marine sediment sample of Jiaozhou Bay, China [36].

Gram-negative bacteria were also found to produce holomycin. Recently, the bioassay-guided isolation has led to the rediscovery of holomycin from a marine Gram-negative bacterium *Photobacterium* *halotolerans* S2753 collected from the southern Pacific Ocean [37]. Furthermore, the fish pathogen *Yersinia ruckeri* was also identified to be a holomycin producer, evidenced through genome-mining, chemical isolation and characterization approaches [38].

In 1969, a new dithiolopyrrolone natural product, antibiotic vD844 (**11**) (Figure 2), was isolated from an unidentified actinomycetes species from a soil sample collected near Copenhagen [14]. Interestingly, antibiotic vD844 has an identical molecular formula and molecular weight to holomycin. Chemical analysis and X-ray finally elaborated that vD844 was 5-oxo-6-(*N*-methylformamido) 4,5-dihydro-1,2-dithiolo[4,3-*b*] pyrrole [14].

The symbiotic bacterium *Xenorhabdus* *nematophilus* XQ1 ATCC 39497 was also found to produce three holomycin derivatives Xenorhabdin 1 (**12**), Xenorhabdin 2 (**13**) and Xenorhabdin 3 (**14**) (Figure 2) [11]. This is the only example among all of the dithiolopyrrolone bacterial producers that produces both of thiolutin-type and holomycin-type natural products, indicating that the *N*-methylation may not be tightly regulated in this organism.

### 2.3. Thiomarinols, PKS/NRPS Hybrid Antibiotic Natural Products

**Figure 3:**
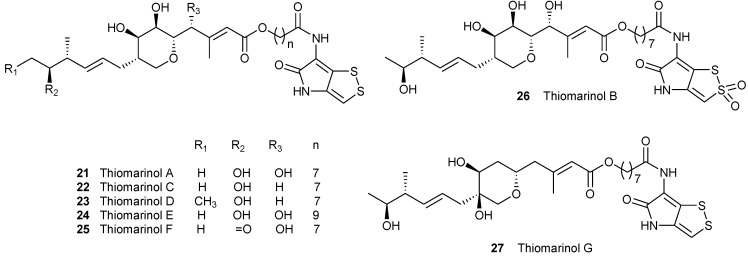
Thiomarinols, hybrid antibiotic natural products.

Thiomarinols (Figure 3) are a unique subgroup of dithiolopyrrolone natural products in that they are hybrid potent antibiotics composed of a dithiolopyrrolone moiety attached via an amid linkage with a pseudomonic acid analogue, an esterified unusual fatty acid component connected with the monic acid, an important polyketide moiety of an antimethicillin resistant *Staphylococcus aureus* (MRSA) antibiotic mupirocin [39,40].

*Pseudoalteromonas* is a genus of marine Gram-negative bacterium. The *pseudoalteromonas* species isolated before 1995 were originally part of the alteromonas genus. *Psudoalteromondas* are known to frequently be bioactive [41] and are often found in association with higher eukaryotes or marine surfaces [42].

In 1993, a Japanese group first reported the fermentation and isolation of thiomarinol A (**21**) (Figure 3) from a marine Gram-negative bacterium *Pseudoalteromonas* sp. nov. SANK 73390 isolated from seawater [16]. Its molecular formula was first established to be C_30_H_44_N_2_O_9_S_2_ with typical UV maxima (300 and 387 nm in methanol) of dithiolopyrrolone chromophore. Further NMR analysis confirmed that the structure of **21** (Figure 3) is a hybrid of two antibiotics, a pseudomonic acid analogue and holothin [43]. Some pseudomonic acid derivatives were also isolated from a marine bacterium *Alteromonas* sp. associated with the marine sponge *Darwinella rosacea* in 1992 [44]. The structure of pseudomonic acid A was identical with that of **21** except for the holothin chromophore moiety in **21**. Soon after this, six new analogues, thiomarinols B–G (**20**–**27**) (Figure 3), were isolated from the same strain [17,18].

Thiomarinol B **20** [17] possesses the same pseudomonic acid component as **21** but differs in the holothin chromophore. The UV spectra and chemical and other spectral properties and X-ray confirmed the presence of a sulfone in the difulfide part of holothin in **26**, rendering that **26** is the only sulfone-containing derivative in the thiomarinol family. The holothin and 7-hydroxyoctanoic acid components of **22**, **23**, **24** and **25** are identical with that of **21** but differ in the modification in the monic acid moiety from **21**. **27** was determined to be 4-deoxythiomarinol A [18]. Compound **23** was found to be 14-homothiomarinol A with one extra methyl in the terminal of the monic acid moiety and **25** to be 13-ketothiomarinol A. **27** is a hybrid of 6-deoxypseudomonic acid B and holothin [18]. Compound **24** is the only thiomarinol derivative containing 8-hydroxynonoic acid moiety but other components of holothin and monic acid are identical to **21**. More recently, six new xenorhabdin derivatives (**15**–**20**) (Figure 3) were also found in the culture broth of *Pseudoalteromonas* SANK73390 with the different chain length of fatty acid component [15].

## 3. Bioactivities and Possible Mode of Action

Dithiolopyrrolone natural products possess broad spectrum of biological activities (Table 2). As one of the first discovered members, thiolutin (**2**) has been extensively studied and found that **2** has a wide range of activities against a variety of Gram-positive and Gram-negative bacteria, protozoa, yeast, pathogenic fungi, and even several human cancer cell lines [45,46,47,48,49,50]. The later discovered that **9** showed similar antibacterial profile to thiolutin [13,29]. Although the structural difference between these two compounds only lies on the methyl group on N4, it is interesting to note that **9** appeared to possess no antifungal activity [13,29]. Thiomarinols are a special group of dithiopyrrolones, which are actually hybrid molecules consisting of one pyrrothine and one psedomonic acid moiety varying in length [16,17,18,51]. Owing to the unique mupirocin-like component in the structure, thiomarinols display much higher activity against *Staphylococcus aureus*, especially the methicillin-resistant *S. aureus* (MRSA), than other dithiolopyrrolones [15].

**Table 2:** Biological activities of dithiolopyrrolones.

Organism		Thiolutin	Holomycin	Thiomarinol
		MIC (μg/mL)/IC_50_ (μM)		
G+	*Bacilius coagulans* CIP 6625	<0.2	NC	NC
	*Bacillus subtilis* ATCC 6633	2	NC	NC
	*Microcoecus leteus* ATCC 9314	<0.2	NC	NC
	*Staphylococcus aureus*	20	4	<0.01
G−	*Klebsiella pneumonia*	1	8	0.78
	*Escherichia coli*	>100	<2	3.13
	*Salmonella enteric*	>100	NC	NC
	*Pseudomanas aeruginosa*	>100	64	0.39
	*Proteus mirabilis*	NC	4	NC
	*Haemophilus influenza*	NC	<0.3	NC
Fungi	*Mucor ramannianus* NRRL 1829	10	NC	NC
	*Penicillium* sp*.*	20	NC	NC
	*Alternaria* sp*.*	20	NC	NC
	*Fusarium*	<40	NC	NC
	*Candida albicans*	20	NC	NC
Yeast	*Saccharamyces cerevisiae*	10	NA	NC
HUVEC	VTN	0.83	NC	NC
	FN	0.16	NC	NC
	COL	0.48	NC	NC

Microbials were tested as MIC and HUVEC were tested as IC_50_ values. NC, unclear; NA, no activity; HUVECs, human umbilical vein endothelial cell; VTN, vitronectin; FN, fibronectin; COL, collagen type IV.

The mode of action for dithiolopyrrolones has been studied to a great extent using **2/9** as the model compounds [29]. It was established that the antibacterial activity of **2/9** against *E. coli* is attributed to the inhibition of RNA synthesis [29,52,53]. However, the dispute of whether **2/9** inhibits the initiation or elongation steps of RNA synthesis has been argued for a long time [52,54]. Khachatourians and Tipper measured the effects of **2** on β-galactosidase expression in *E. coli*, and suggested that this compound inhibits RNA chain elongation [52]. In contrast to this conclusion, the study performed by Sivasubramanian and Jayaraman indicated that **2** inhibits initiation of RNA transcription [54]. To resolve the above discrepancies, the mode of action of dithiolopyrrolones was reinvestigated using holomycin as the model [50]. By characterizing the effects of **9** on the kinetics of β-galactosidase expression, Oliva *et al.* confirmed that **9** inhibits RNA polymerase at the level of RNA chain elongation rather than initiation [50]. More supportive evidence comes from a study characterizing activities of various RNA polymerase inhibitors against *Staphylococcus aureus* mutants that display resistance to rifampin, an inhibitor of transcription initiation. O’Neil *et al.* found that both **2** and **9** are both active against *S. aureus* strains containing mutant RNA polymerase β-subunit (*rpoB*) gene that confers resistance to rifampin [55]. This result suggested that the target site(s) of dithiolopyrrolones is different from that of rifampin, and dithiolopyrrolones only affect mRNA transcription at the phase of elongation. Recently, an RNA methyltransferase Hom12, which can methylate the RNA and hence protect the host from the cytotoxic effect of **9**, was characterized in the **9** producing fish pathogen *Yersinia ruckeri* [38]. This study proposed that RNA methylation may interfere with the activity of RNA polymerase by **9**, consistent with the finding that the mutant *E. coli* strain harboring *hom12* showed tolerance to **9**. Future studies on the exact RNA substrate of Hom12 and the relationship between such RNA species and RNA polymerase will shed light on the *in vitro* reconstitution of the mode of action of holomycin, and therefore the whole dithiolopyrrolone family.

The mechanism underlying the inhibition of RNA polymerase by dithiolopyrrolones still remains to be revealed. However, the structural characteristics of dithiolopyrrolone core scaffold, the disulfide-bridged heterocycle, may give some hints to this question. The mycotoxin gliotoxin and the histone deacetylase inhibitor FK228 are two compounds that possess a similar disulfide bond [38,56]. It was shown that the activities of these molecules are due to the reduction of the disulfide bond in the cell, giving rise to the more active dithiol groups which can react with target proteins' thiol groups [56,57]. By analogy, dithiolopyrrolone compounds may behave in the same way to inhibit RNA polymerase. In support of this hypothesis, Li *et al.* found that there were a number of intermediates, with the dithiol groups modified by a combination of mono- and di-*S*-methylation, accumulating in a mutant holomycin producer, in which the gene (*hlmI*) responsible for the disulfide formation was deleted [58]. This result suggested that the dithiol intermediates produced by *ΔhlmI* mutant may be very active even toxic, and the host can protect itself by incapacitating the reactive dithiol groups. Beside the above "reduction" mechanism concerning dithiolopyrrolone action mode, an “oxidation” mechanism was also proposed. Juhl *et al.* found that *E. coli* strains carrying the *thdA* (sulfone oxidase) mutation showed hypersensitivity to thiolutin. Since these *thdA* mutants possess high oxidation activities toward a wide variety of substrates containing sulfur, the authors implied that oxidation of thiolutin may induce its toxicity in the cell [59].

RNA polymerase represents an attractive target for the development of high-efficiency antibacterial drugs because transcription is essential for bacterial growth and survival [60]. So far, the class of rifamycins is the only clinically used natural RNA polymerase inhibitor [61]. However, with the emergence of rifamycin-resistant bacteria that even possesses cross-resistance to the other RNA polymerase inhibitors, the development of new drug candidates that have different target sites from rifamycins is in demand [60,61]. Dithiolopyrrolone class of compounds, such as **2** and **9**, could be considered to be the warhead for designing the next-generation of RNA polymerase-associated drugs. Yakushiji *et al.* recently developed a series of novel bacterial RNA polymerase inhibitors by incorporating holomycin into several myxopyronin skeletons [57]. One of the resulting compounds exhibits good antimicrobial activity against Gram-positive bacteria, implying that using the pyrrothine as a component to make hybrid-type drugs is a promising direction for novel drugs development.

## 4. Total Synthesis of Dithiolopyrrolones

Total syntheses of dithiolopyrrolones have been attempted since the early 1960s, and many synthetic strategies have been developed.

**Scheme 1:**
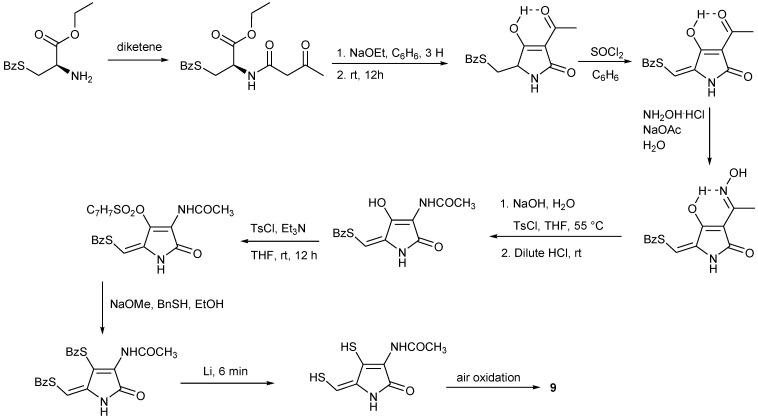
Lukas’s synthesis of holomycin.

**Scheme 2:**
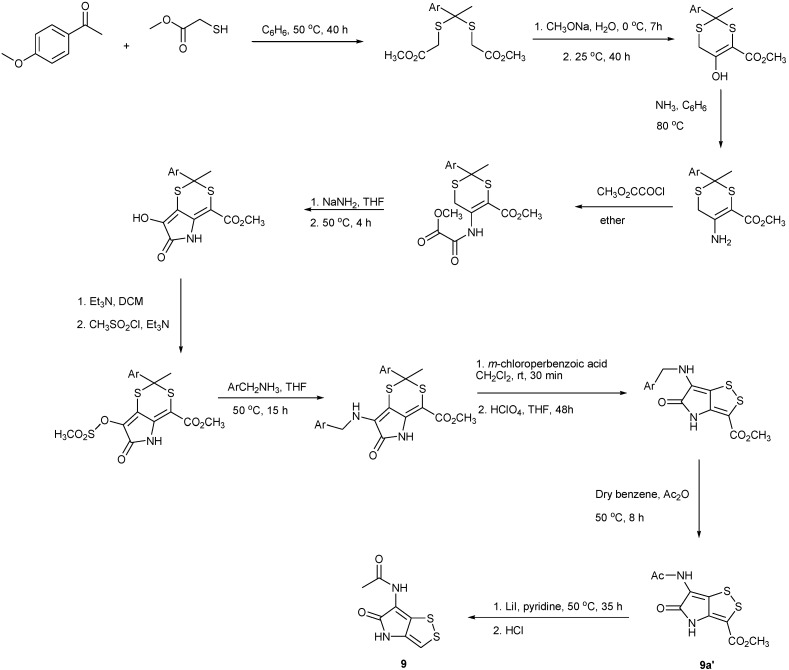
Ellis’s synthesis of holomycin (**9**) and its carboxylated derivative (**9a’**).

The first total synthesis of thiolutin (**2**) and derivatives was achieved in 1962 starting with *N*-methyl-1-ethoxycarbony l-2-diethoxyethylamine and methoxycarbonylacetyl chloride [62]. In 1964, Lukas and Buchi proceeded along a different synthetic route with the starting material of *S*-benzyl-l-cysteine ethyl ester but through the same dithiol intermediate as reported in 1962 (Scheme 1) [63]. These syntheses of **9**, however, have relied on the oxidation of the common intermediate, reduced dithiolopyrrolone dithiols, to create the disulfide ring and have not been adaptable to the preparation of ring-substituted derivatives [64]. Later on, Ellis *et al.* devised the synthesis of the preparation of holomycin and its 3-carboxylated derivative starting with *p*-methoxyacetophenone and methyl thioglycolate (Scheme 2) [65]. Among these 10-stage synthetic steps, highlighted were the two key reactions, construction the substituted pyrrolinone ring by cyclization of the methoxalylamine and contraction of the 6-membered dithioketal to the 5-membered disulfide ring of 3-carboxyholomycin adapted from the method developed by Kishi and co-workers [66]. Holomycin (**9**) was finally obtained in a single step by cleavage and concomitant decarboxylation from 3-carboxyholomycin **9a’** (Scheme 2) [65].

**Scheme 3:**
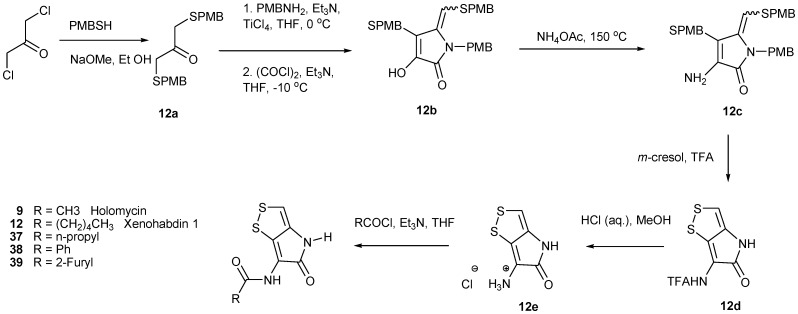
Hjelmgaard’s synthesis of holomycin and its derivatives.

Recently an efficient and convenient synthetic route has been developed for the preparation of **9**, xenorhabdin I (**12**) and some other analogs thereof (Scheme 3) [67]. The reaction started with 1,3-dichloroacetone by treatment of *p*-methoxybenzylthiol (PMBSH) to yield the **12a** in a one-pot procedure. The amine functionality was next introduced by reaction of **12b** with ammonium acetate to give **12c** in 87% yield. TFA-pyrrothine **12d** was generated by refluxing **12c **in TFA in the presence of *m*-cresol in order to remove the PMP protecting groups and simultaneously form the pyrrothine skeleton. The benefit of this synthetic route would give fast access to an intermediate pyrrothine with a free amino, which would ease analog synthesis. Another highlight in this contribution was that this method used *p*-methoxybenzyl (PMB) group instead of *t*-butyl group as protective group which requires the use of toxic and environmentally hazardous mercuric acetate for removal (Scheme 3) [67].

**Scheme 4:**
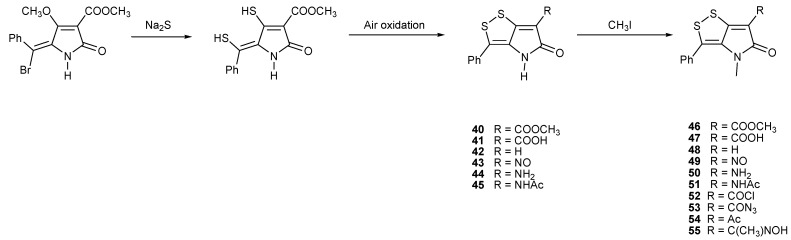
Stachel’s synthesis of dithiolopyrrolones.

The downside of the methods presented on Scheme 1, Scheme 2 and Scheme 3 are the relatively low yields and lack of versatility in providing various derivatives needed for biological studies. Stachel and co-workers demonstrated modified and versatile synthetic routes of preparation of ring-fused dithiinopyrroles, dithiolopyrroles and pyrroloisothiazoles [68]. A series of phenyl-substituted dithiolopyrrolones were prepared starting from the known lactam pyrrolinone. The key reaction was based on the nucleophilic displacement of the methoxy and bromine by Na_2_S, giving a dithiolate; the latter was readily oxidized by O_2_ in air forming dithiolopyrrolones. Furthermore, the *N*-methylated derivatives were obtained by reacting with MeI (Scheme 4) [69].

**Scheme 5:**
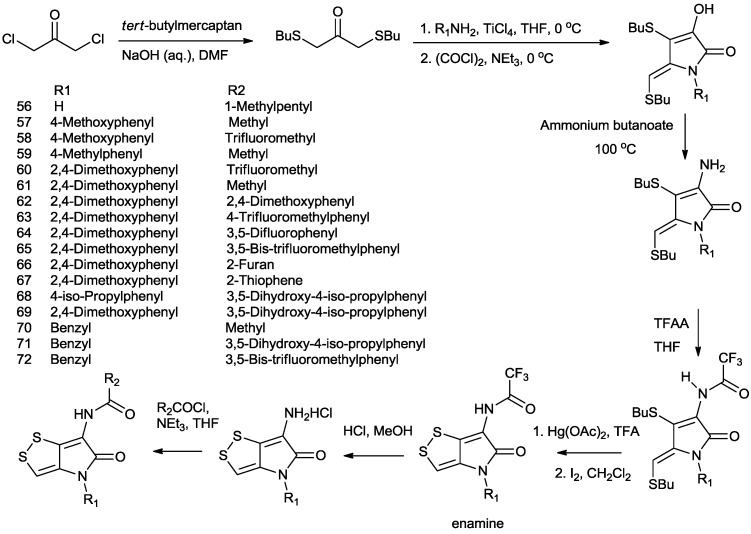
Li’s synthesis of dithiolopyrrolone derivatives.

Li *et al.* developed an expedient manner (seven steps) of the total synthesis of dithiolopyrrolones from commercially available starting materials in a kilogram scale and prepared 17 of dithiolopyrrolone derivatives with aromatic substituents on the pyrrolone nitrogen atom (Scheme 5) [70,71]. The key step for introduction of *N*-substituted aromatic group was the reaction of ketone intermediate with the appropriate aromatic primary amines in tetrahydrofuran to afford the cyclic enols in good yield (60%–70%), followed by the conversion into the corresponding cyclic enamines. The remaining steps towards the synthesis of pyrrolones were considerably similar to the ones previously reported [67].

Gao and Hall *et al.* reported the first total synthesis of a thiomarinol derivative with three components, pseudomonic acid, 8-hydroxyoctonoic acid and anhydroornithine [72]. The total yield after 13 synthetic steps was 22%. The concise synthetic route of a stereoconvergent three-component strategy was considered to be amendable to the design of other analogues, *i.e.*, thiomarinol A.

## 5. Biosynthesis of Dithiolopyrrolones

### 5.1. Precursor-Directed Biosynthesis (PDB) of Dithiolopyrrolones

The generation of natural product analogues is often important for improving bioavailability to fine tune compounds’ activity [73]. PDB has proven to be a powerful tool for the synthesis of structural analogues [74]. PDB takes advantage of the natural flexibility of biosynthetic pathways toward the acceptance of unnatural precursor analogues. Analogs of biosynthetic building blocks are designed, synthesized and fed to the organism and the biosynthetic enzymes, if in degree of promiscuity, then incorporate this unnatural building block into the natural product so that analogs of natural product of interests will be generated [74].

**Figure 4:**
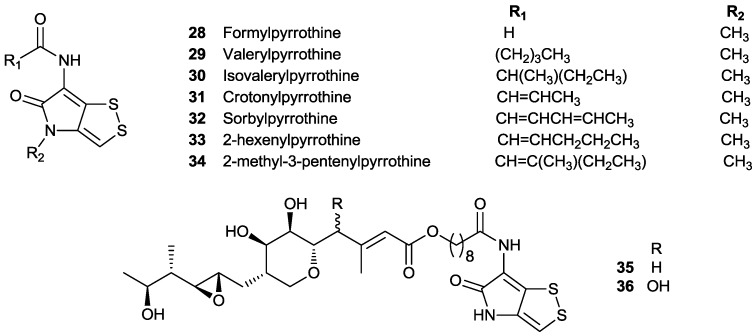
“Unnatural” dithiolopyrrolone natural products using precursor-directed biosynthesis.

*Saccharothrix algeriensis* is a rare actinomycete isolated from the soil of the palm groves of Southern Algeria [9]. Five thiolutin-type dithiolopyrrolones with different branched chains and chain length of acyl groups were obtained from the fermentation broth of *S. algeriensis*, implying that there may have some degree of plasticity for the enzymes responsible for bioconversion of organic acid into acyl-CoA and installation of acyl-CoA into the holothin skeleton. Bouras *et al.* then explored this property by introduction of various organic acids into fermentation media. The addition of only three acids, benzoic, valeric and cinnamic acids, led to the production of unnatural dithiolopyrrolones identified in the culture broth of *S. algeriensis* [24]. Of particular interest was the incorporation of aromatic acids into the scaffolds of dithiolopyrrolones, indicating the enzyme promiscuity in the biosynthetic pathway of dithiolopyrrolones in *S. algeriensis* (Figure 4). Adding valeric acids into the fermentation medium of *S. algeriensis* also induced the production of three new antibiotic dithiolopyrrolones, formylpyrrothine **28**, valerylpyrrothine **29** and isovalerylpyrrothine **30** [75]. Further exploitation of PDB method led to identification of four new dithiolopyrrolone antibiotics, crotonylpyrrothine **31**, sorbylpyrrothine **32**, 2-hexenylpyrrothine **33** and 2-methyl-3-pentenylpyrrothine **34**, in the presence of 5 mM sorbic acid in the production medium, showing the remarkable flexibility of the dithiolopyrrolone biosynthetic pathway in *S. algeriensis* [76].

Recent genome sequencing of the thiomarinol producer bacterium *Pseudoalteromonas* sp*.* SANK 73390 indicated that thiomarinols are biosynthesized from two independent pathways, an AT-less type I PKS one for marinolic acid and a NRPS one for holothin [77]. Inactivation of one of domains in PKS genes resulted in the PKS mutant in which the production of thiomarinols was completely abolished. Feeding pseudomonic acid A (0.1 mg mL^−1^) immediately after inoculation resulted in identification and isolation of two new derivatives, a pyrrothine derivative of pseudomonic acid **35** and its 4-hydroxylated analogue **36** along with three derivatives of pseudomonic acid A (Figure 4) [15].

### 5.2. Biosynthesis of Dithiolopyrrolones

Despite the emerging importance of dithiolopyrrolones, the dearth of the biosynthetic knowledge was particularly striking until recently. The difficulty to locate constituent gene segments even if they were clustered may result from the unusual heterobicyclic and highly oxidative dithiolopyrrolone skeleton. Early feeding experiment demonstrated that l-cystine appeared to be the precursor of dithiolopyrrolone biosynthesis and that pyrrothine seemed to be an intermediate in the pathway from l-cystine to dithiolopyrrolone [78,79].

It has been speculated that an *N*-acetyltransferase type of enzyme could be involved in the late stage of the holomycin biosynthesis. Indeed, the presence of such an enzyme appeared to be necessary for the amide bond formation between the holothin nucleus (deacetylholomycin) and acetylCoA in cell-free extracts of the holomycin-overproducing mutants of *S. clavuligerus* [80]. A similar result was also shown that incubation with *N*-methylpyrrothine and acetylCoA or benzoylCoA in the cell-free extract of *S. algeriensis* NRRL-24137 resulted in formation of thiolutin or *N*-methyl-*N*-benzoylpyrrothin, respectively [81].

#### 5.2.1. Identification of the Holomycin Gene Cluster in *S. clavuligerus*

Analysis of *Streptomyces clavuligerus* genome sequence indicated that *S. clavuligerus* has a relatively small chromosome of 6.8 Mb in length but contains a megaplasmid of 1.8 Mb in length [82]. There are 48 putative secondary metabolite gene clusters that have been identified by a homolog comparison. Among these gene clusters, 23 are in the chromosome and 25 are in the megaplasmid. Taken advantage of the genome mining strategy, the holomycin biosynthetic gene cluster in *S. clavuligerus* has recently been identified and characterized, as evidenced through heterologuous protein expression, enzyme activity assays [83] and heterologuous expression of the gene cluster [84,85].

**Figure 5:**
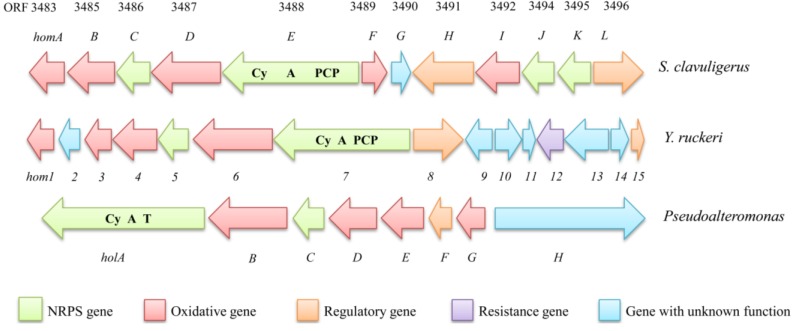
Comparison of the genetic organization of the holomycin biosynthetic gene clusters from *S. clavuligerus*, *Y. ruckeri* and *Pseudoalteromonas*, respectively.

The holomycin gene cluster consists of 12 genes, spanning an approximately 17.6 kb region in the chromosome of *S. clavuligerus*, ten of which the functions have been assigned (Figure 5, Table 3) [83,84]. The gene cluster only contains a gene (*orf*3488 [83] and *homE* [84]) encoding a multidomain non-ribosomal peptide synthetase (NPRS) with a conical order of cyclization (Cy), adenylation (A) and thiolation domains (T). Of particle interest is that the gene cluster also encodes four flavin-dependent oxidoreductases (ORF3483, 3487, 3489, 3492 [83] or HomB, D, F, I [86]) and a putative acetyltransferase (ORF 3484 [83] or HomA [84]). Additionally, three stand-alone NRPS encoded proteins were found in the gene cluster. These are freestanding C domain (ORF3495 [83] or HomK [84]), the Te Domains (ORFs 3486 and 3494 [83] and Hom*C* and HomJ [84]). Two genes in the cluster, *orf* 3491 and 3496 (*hom*H and *hom*L [84]), respectively, were predicted to be a regulatory gene and transporter gene, respectively.

**Table 3:** Deduced functions of open reading frames (ORFs) that were predicted to be involved in the biosynthesis of holomycin in *S. clavuligerus, Y. ruckeri* and *Pseudoalteromonas*, respectively.

ORFs in *S. clavuligerus* [83]	Homolog in *Y. ruckeri* (Identity %) [38]	Homolog in *Pseudoalteromonas* (Identity %) [87]	Proposed Function
ORF3489(HlmF)	Hom1 (61%)	HolG (72%)	PPC-DC decarboxylase
ORF3490(HlmG)	Hom2(65%)	HolF (70%)	Globin
ORF3483(HlmA)	Hom3 (38%)	HolE (45%)	*N*-acyltransferase
ORF3485(HlmB)	Hom4 (58%)	HolD (63%)	Acyl-CoA dehydrogenase
ORF3486(HlmC)	Hom5 (36%)	HolC (42%)	Thioesterase
ORF3487(HlmD)	Hom6 (47%)	HolB (59%)	FMN-dependent oxdioreductase
ORF3488(HlmE)	Hom7 (47%)	HolA (55%)	NRPS (Cy-A-T)
ORF3491(HlmH)	Hom8 (61%)		MFS efflux protein

Gene disruption of *orfs* 3488 and 3489 in the holomycin-overproducing mutant completely abolished holomycin production, indicating that the identified gene cluster is responsible for holomycin production [83]. We also demonstrated that introduction of the whole gene cluster into a heterologuous host *Streptomyces albus* resulted in the production of holomycin in the mutant *S. albus* [84].

#### 5.2.2. Characterization of Key Enzymes during the Holomycin Biosynthesis in *S. clavuligerus*

Given that genetic evidence demonstrated the involvement of ORF3488 for the holomycin production, it was overproduced in *E. coli*. The amino acid-dependent exchange assay showed that the adenylation domain of ORF3488 proceeds aminoacylation of l-cysteine but not the other proteinogenic amino acid with a *K_m_* value of 1 mM and a *K_cat_* value of 98 min^−1^ [83].

**Scheme 6:**
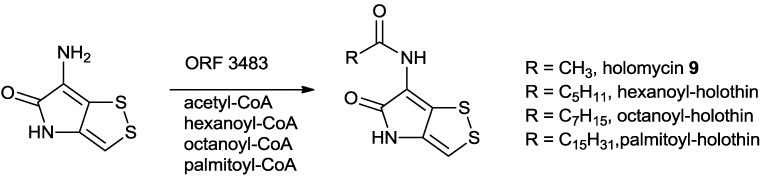
Biochemical study confirmed that the *N*-acetyl-CoA transferase ORF3483 is responsible for the amid bond formation at the late stage of the holomycin biosynthesis in *Streptomyces clavuligerus*.

The predicated activity of the encoded ORF3483 was an *N*-acetylCoA transferase. Incubation of recombinant ORF3483 (10 nM) with acetylCoA and holothin (20 nM) showed the formation of holomycin with an apparent *K_m_* of 6 nM and a *K_cat_* of 80 min^−1^, reassuring the involvement of ORF3483 during the biosynthesis of holomycin (Scheme 6). Surprisingly, recombinant ORF3483 was also able to utilize longer chain acyl CoAs (hexanoyl, octanoyl and palmitoylCoA) as substrates with less efficiency. The apparent *K_m_* of 30 nM and apparent *k_cat_* of 0.07 min^−1^ was obtained from octanoylCoA in the presence of 20 nM holothin [83]. Longer chained acyl holothins were not observed in the fermentation broth of *S. clavuligerus* presumably because the pools of these fatty acids or acylCoAs could be very low. Identification of longer acyl chain variants of dithiolopyrrolones, however, was observed in other microorganisms [9,16].

It is rare for molecular scaffolds of bacterial natural products to contain disulfide bonds, and the mechanism of disulfide bond formation in these products is poorly understood until recently [86]. The first evidence of the disulfide bond formation was reported in 2009 during the study of the biosynthesis of FK228, a disulfide-containing anticancer despeptide natural product isolated from the soil bacterium, *Chromobacterium violaceum* 968. The identified enzyme DepH represents a new subclass of the thioredoxin protein superfamily [86]. In 2010, another homolog enzyme GliT was found to be responsible for the disulfide-bond formation in the biosynthesis of Gliotoxin, a disulfide-containing metabolite isolated from the human pathogen *Aspergillus fumigatus* [88]. Although both DepH and GliT belong to a new member of FAD-dependent dithiol oxidases, DepH utilizes NADP^+^ as the electron acceptor [86] while GliT use O_2_ to promote the disulfide formation [88].

**Scheme 7:**
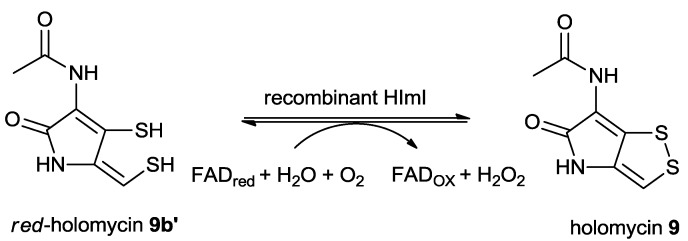
Biochemical study confirmed that the dithiol oxidase HlmI is responsible for the disulfide bond formation using molecular oxygen as a cofactor.

*In silica* analysis of the holomycin gene cluster in *S. clavuligerus* showed that the encoded flavoenzyme HlmI [89] (ORF3492 [83] and HomI [84]) may function analogously to DepH or GliT to convert dithiol form of reduced holomycin **9b’**/holothin **9b** into holomycin **9**/holothin **9a** (Scheme 7). Indeed, incubation of purified recombinant HlmI (50 nM) with FADH_2_ and reduced holomycin **9b’** (5–100 nM) led to the rapid formation of holomycin in presence of oxygen with an apparent *K_m_* of 4.6 ± 1.9 nM and an apparent *k_cat_* of 333 ± 28 min^−1^. Although HlmI clearly accelerated the disulfide bond formation from reduced holothin **9b** to holothin **9a**, the nonenzymatic oxidation in presence of oxygen precluded kinetic measurement [89]. It was concluded that HlmI is a GliT-like FAD-dependent dithiol oxidase, using O_2_ as the oxidative agent for the formation of intramolecular disulfide bridges in the late stage of the holomycin biosynthesis [89].

#### 5.2.3. Regulation of the Biosynthesis of Holomycin in *S. clavuligerus*

Regulation of the holomycin production in *Streptomyces* appeared to be very complex. Early studies indicated that holomycin production appeared to be associated with the production of cephamycin C. For example, the wild type *Streptomyces* sp. P6621 produces cephamycin C but does not produce **9**. Chemical mutagenesis of *Streptomyces* sp. P6621 resulted in the production of **9** and the reduced yield of cephamycin C [32]. The wild type *S. clavuligerus* only produces trace amount of holomycin. UV mutagenesis led to generate the mutant IT1 that was a holomycin-overproducing strain [29]. In 2001, Liras *et al.* demonstrated that gene knockout in the gene cluster of clavulanic acid in *S. clavuligerus* resulted in overproduction of holomycin, suggesting that the intriguingly intricate cross-regulation between the biosynthetic pathways of clavulanic acid and holomycin [80].

A rhodanese-like protein was found to be highly overrepresented in the proteome of the holomycin-overproducing mutant of *Streptomyces clavuligerus.* Disruption of the rhodanese-like gene resulted in great loss of holomycin production in the *rhlA* mutants [90].

Addition of arginine appears to stimulate the production of holomycin [91,92]. The gene *argR* is a universally conserved repressor gene in the arginine biosynthesis in S*. clavuligerus* NP1. Disruption of *argR* resulted in holomycin-overproducing mutant, *S.* *clavuligerus* CZ [93]. Comparative proteomic studies demonstrated that the expression levels of proteins involved in acetyl-CoA and cysteine biosynthesis increased in the mutant CZR strain, consistent with the holomycin overproduction phenotype [93].

The genes *afsR* and *afsS* in *S. clavuligerus* ATCC27064 encode proteins resembling the well-known antibiotic biosynthetic activators. It was found that re-introduction of *afsRS_cla_* genes into the wild-type *S. clavuligerus* activated the normally silent holomycin biosynthetic gene cluster while the production of clavulanic acid was also increased 5-fold in resultant mutant compared to the wild-type strain [94].

A competition-based adaptive laboratory evolution could accelerate the discovery of antibiotics when an antibiotic-producing microorganism is competed against a drug-resistant pathogen [95]. Of particular interest is that actinomycetes that are well known producer of secondary metabolites could adaptively evolved in the laboratory to produce new antibacterial compounds, of which the production is silent in the normal laboratory culture conditions [96]. Palsson *et al.* demonstrated that, after several rounds of co-culturing *S. clavuligerus* and the methicillin-resistant Staphylococcus aureus (MRSA) N315, a mutant strain of *S. clavuligerus* emerged that acquired the ability to constitutively produced holomycin, the antibacterial agent that inhibits the growth of MRSA [97]. Genome sequencing revealed that the mutant strain had lost the megaplasmid, and acquired genetic mutations that affected secondary metabolite biosynthesis [97].

More recently, RT-PCR transcription analysis of the holomycin-overproducing mutant of *S. clavuligerus* showed a higher transcription of some genes in the holomycin gene cluster compared with the ones in the wild-type strain [85]. This result was consistent with the proteomic analysis of the holomycin overproducer mutant that some transcribed proteins related to the holomycin pathway were overexpressed [85].

#### 5.2.4. Identification of the Holomycin Gene Cluster in the Fish Pathogen *Yersinia ruckeri*

Homolog search indicated that several open reading frames (ORFs) in the genome of the fish pathogen *Yersinia ruckeri* appear to be homologous to the ones in the holomycin pathway of *S. clavuligerus*, including three oxidoreductases, one thioesterase and, more importantly, one multidomain NRPS with a conical order of Cy-A-T arrangement. However, this gene cluster lacks two key homolog genes, one encoding the dithiol oxidase that promotes the disulfide bridge formation and the other encoding the freestanding condensation domain. Our chemical isolation and structural elucidation demonstrated that *Y. ruckeri* is a producer of holomycin. Gene disruption of *hom*6, a homolog of homD [84], completely abolished the production of holomycin in the mutant strain, suggesting that the identified gene cluster directs the biosynthesis of holomycin.

#### 5.2.5. The Proposed Mechanism of the Formation of Holomycin

**Scheme 8:**
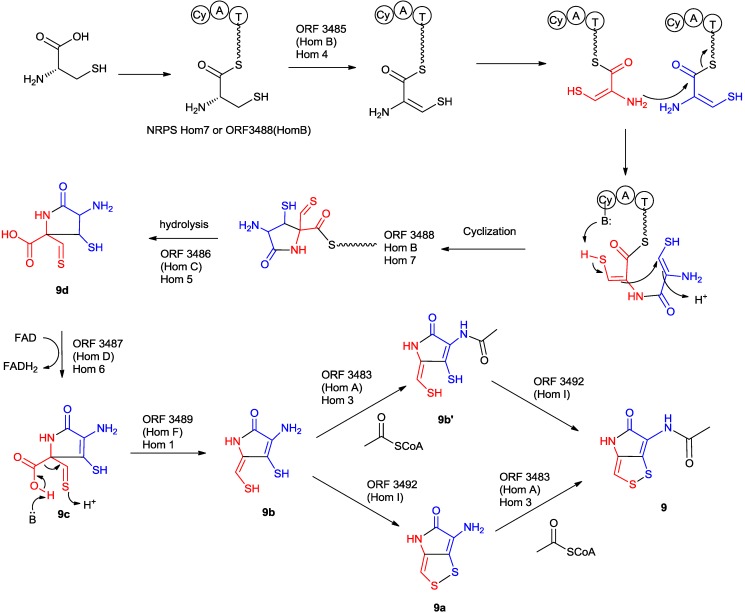
Proposed biosynthetic pathway of holomycin.

Despite the differences between two holomycin gene clusters from the Gram-positive bacterium *S. clavuligerus* and the Gram-negative bacterium *Y. ruckeri*, the underlying chemical logic of holomycin formation should be similar (Scheme 8).

Biochemical and genetic evidence demonstrated that the formation of holomycin should follow the same chemical logic as other biosynthetic pathways of non-ribosomal peptides in which a tridomain non-ribosomal peptide synthetase (HomE [84] or HlmE [83] or Hom7 [38]) first selects and activates l-cysteine. The condensation activity was proposed to follow an unusual pathway [98]. In *S. clavuligerus*, it was proposed that the flavin-dependent acyl-CoA dehydrogenase (HomB [84] or HlmB [83]), the standalone C domain and the Cy domain of the NRPS are responsible for oxidizing, coupling, and cyclizing two cysteine residues to yield a cyclodithiol-PCP-domain tethered intermediate **9f**. In *Y. ruckeri*, no dedicated C domain can be found within the holomycin gene cluster. Thus the Cy domain may have dual functions that catalyze both condensation and cyclization of C-C formation, although it clearly remains speculative until the studies of the detailed mechanism is carried out [38].

The cyclodithiol-PCP-domain tethered intermediate **9f** could then be hydrolyzed by the thioesterase (HomC [84] or HlmC [83] or Hom5 [38]) to generate the free acid intermediate **9e**. The glucose-methanol-choline oxidoreductase homolog (HomD [84] or HlmD [83] or Hom6 [38]) could be responsible for the 2-electron oxidation step on **9d** into **9c**. HomF [84] (HlmF [83] or Hom1 [38]) is an analog of phosphopantothenoylcystein decarboxylase in coenzyme A biosynthesis. In the reaction cycle of PPC-DC, the thiol moiety of pantothenoylcysteine is first oxidized and spontaneously decarboxylatedto generate the pantothenoylaminoethenethiol intermediate, which is finally reduced to form pantothenoylcysteamine. In analogy, HomF [84] (HlmF [83] or Hom1 [38]) could catalyze the decarboxylation of the intermediate **9c** into **9b**. The assignment does not, however, suggest a preferred sequence for these activities [83]. In *S. clavuligerus*, HlmI appeared to play important roles in the biosynthesis of holomycin. Li *et al.* has confirmed that recombinant HlmI mediates the disulfide bond formation from reduced holomycin **9a** to holomycin **9** using O_2_ as cofactor, and it was proposed that HlmI is involved in the late stages of holomycin biosynthesis [89]. Gene disruption of *hlmI* resulted in decreased production of holomycin and increased sensitivity toward holomycin [89]. The homolog of HlmI, however, cannot be found in the holomycin gene cluster in *Y. ruckeri* and a similar absence was also observed in the thiomarinol gene cluster from *Pseudoalteromonas* sp. SANK73390, indicating the different underlying chemical logic of disulfide bond formation in Gram-negative bacteria [38]. Biochemical evidence demonstrated that HlmA is responsible for the acylation of the amino group in holothin **9b** or reduced holothin **9a** [83].

### 5.3. Biosynthesis of Thiomarinol Natural Products

Thiomarinols belong to a special group of dithiolopyrrolones in that they are hybrid antibacterial compounds consisting of three components, a pseudomonic acid moiety esterified by a terminal-hydroxy fatty acid (*n* = 7 or 9) attached to the holothin moiety via an amide linkage.

Recently genome sequence of the thiomarinol-producer bacterium revealed a novel plasmid, pTML1 with the length of 97 kbp [87]. Interestingly, the plasmid contains two distinct gene clusters, one responsible for the biosynthesis of pseudomonic acid and the other for the holothin moiety. The pseudomonic acid gene cluster contains the typical feature of *trans*-AT/AT-less polyketide synthase (PKS) assembly line in that the encoded multidomain PKSs do not contain dedicated acyltransferase domain to activate the acyl substrate. The gene cluster for holothin moiety is similar to the one in *Y. ruckeri*, consisting of 7 genes encoding a multidomain NRPS HolA (Cy-A-T, homolog to Hom7), an oxidoreductase HolB (homolog to Hom6), a thioesterase HolC (homolog to Hom5), a dehydrogenase HolD (homolog to Hom4), a *N*-acyltrasferase HolE (homolog to Hom3), an flavin-dependent oxygenase HolF (homolog to Hom2) and a decarboxylase HolG (homolog to Hom1), respectively (Figure 5). It appeared that the chemical logic for holothin scaffold during the biosynthesis of thiomarinols should be the same as the one of holomycin. Inactivation of *holA* resulted in completely loss of thiomarinol but the only production of marinolic acid in the mutant strain [77], confirming that the *hol* gene cluster is responsible for the holothin biosynthesis, and marinolic acids and dithiolopyrrolones are biosynthesized from two independent pathways.

**Scheme 9:**
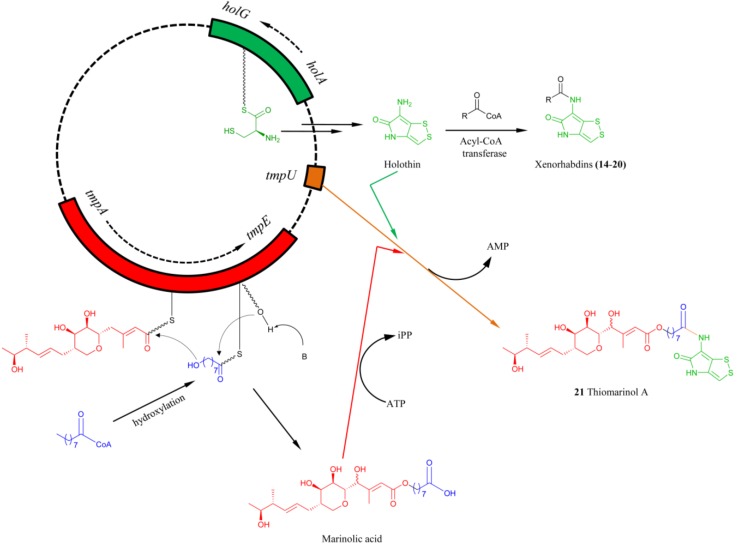
Proposed biosynthetic pathway of thiomarinol A.

In the late stage of the holomycin biosynthesis from both *S. clavuligerus* and *Y. ruckeri*, acyl-CoA was proposed to be the substrate of the acyl CoA transferase that mediates the amide bond formation for the holomycin production. In the thiomarinol biosynthesis, TmlU was assigned as an ATP-dependent ligase, a homolog of SimL in the simocylinone biosynthesis [77,99] and NovL in the novobiocin biosynthesis that catalyze the amide bond forming activity with a variety of carboxylic acids [100]. Inactivation of *tmlU* completely abolished the production of thiomarinols but resulted in the production of xenorhabdins and marinolic acids, pseudomonic acid derivatives, thus suggesting its role of linking the pseudomonic acid and holothin to generate thiomarinols. The production of xenorhabdins and derivatives, however, indicates that HolE, a homolog of acylCoA transferase, could be the second copy of amide-formation enzyme responsible for the installation of acylCoA into the amino group of holothin to generate xenorhabdins **14**–**20** (Scheme 9).

## 6. Conclusions

The family of dithiolopyrrolone natural products has attracted attention from the research communities of natural product chemistry/biosynthesis, synthesis and microbiology on their unique chemical identity and multiple biological activities. There have been challenging questions of the biosynthesis, the complex regulation network and the mode of action of this novel class of molecules during the last decade. Recent efforts on the biosynthetic pathways of holomycin and thiomarinols have just started to uncover the intriguing aspects of the underlying chemical logic, regulation and resistance of this class of molecules. This review article has covered the natural product discovery, synthesis, bioactivity and biosynthesis of this class of natural products in the first time over sixty years. Further progress in this class of molecules will be to understand the biochemistry of the formation of the pyrrolone chromophore and the timing of *N*-methylation in thiolutin-type of molecules, and to ascertain the exact antibacterial mode of action, which will facilitate a greater understanding of this promising class of antibacterial and antitumor agents.
